# *Ex-vivo* biomechanical evaluation of the application of a novel annulus closure device to closure of annulus fibrosus

**DOI:** 10.3389/fbioe.2024.1337269

**Published:** 2024-06-04

**Authors:** Yijian Ying, Kaiwen Cai, Xiongxiong Cai, Kai Zhang, Rongzhang Qiu, Hangtian Hu, Guoqiang Jiang, Kefeng Luo

**Affiliations:** ^1^ Health Science Center, Ningbo University, Ningbo, China; ^2^ Department of Orthopaedics, The First Affiliated Hospital of Ningbo University, Ningbo, China

**Keywords:** annulus fibrosus closure, suture, functional discal units, minimally invasive, discectomy

## Abstract

**Objective:**

To investigate the technical feasibility of applying a simple suture guide device to close the annulus fibrosus (AF) of the intervertebral discs (IVD).

**Methods:**

30 sheep functional discal units (FDUs) were obtained and subjected to mock discectomy. Mock sutures were performed using 3–0 non-absorbable sutures under a novel AF suture device following a suture procedure. The FDUs were compressed under axial loading at 1.8 mm/min and evaluated for Failure load (N).

**Results:**

The failure loads of the hand stitching group (Group H) and suture device stitching group (Group S) were significantly higher than those of the control group (Group C) (*p* = 0.033; *p* < 0.001).

**Conclusion:**

This study provides reasonable reasons to believe that the simple suture guide device described here is technically feasible for AF defect closure. It thus constitutes an encouraging proof of concept for the proposed device; however, it does not constitute a complete demonstration of the device’s feasibility in the clinical setting considering that the annulus closure operation is performed *ex vivo* on functional spinal units, as opposed to within an environment that mimics the clinical setting. To this end, confirmatory experiments will be conducted such as more multiaxial or dynamic mechanical testing, and notably performing the surgery on sheep models instead of on *ex vivo* functional spinal units.

## Introduction

Lumbar disc herniation (LDH) is one of the leading causes of low back pain (LBP) (Morlion, 2013; Risbud and Shapiro, 2014; Hartvigsen et al., 2018). LBP dramatically reduces the quality of patients’ lives, as well as posing a significant socio-economic burden (Hoy et al., 2014; Dowdell et al., 2017).

The recurrence rates have been reported to be 0%–15% for conventional nucleus pulposus (NP) removal surgery (Moliterno et al., 2010; Casal-Moro et al., 2011; Matsumoto et al., 2013; Gibson et al., 2017; Zhang et al., 2018) and 0.8%–11% for microendoscopic or foraminoscopic procedures (Choi et al., 2015; Yin et al., 2018; Park et al., 2019), both of which have high recurrence rates. Surgical treatment relieves nerve root compression by eliminating herniated tissue. However, low cellularity, non-vascularity (Vernon-Roberts et al., 2007), and poor regenerative capacity (Bailey et al., 2013) of the AF lead to a high incidence of post-surgical reherniation (Andersson, 1999; Bailey et al., 2013), potentially causing adjacent vertebral degeneration (Häkkinen et al., 2007). Patients with significant AF defects (≥6 mm) after lumbar discectomy were found to be at higher risk of symptomatic recurrence and reoperation (Miller et al., 2018). In summary, the recurrence rate of LDH is high and dramatically reduces the postoperative quality of life of surgical patients. Effective closure of AF breaks is essential.

However, Not all herniation procedures require AF closure, and the application scenario for the suture technique is in patients who are considered to have a high recurrence rate in the preoperative evaluation (e.g., young and middle-aged manual workers, patients with abundant disc content and a large intervertebral space, and patients with intervertebral space instability). Regarding whether reherniation occurs after AF closure, Suh et al. found no recurrence at 3 years after AF closure in 19 patients (Suh et al., 2015). Of course, there is also a risk of failure in AF closure. Gauthen et al. studied 254 surgical patients with LDH and found that the 2-year recurrence rate was 21% in patients without AF sutures, 10% with 1-stitch sutures, and decreased to 5% with 2-stitch sutures (Gauthen, 2005).

Annulus closure devices (ACDs) such as Xclosure, Barricaid annular closure devices, etc., have been launched, and the clinical outcomes are encouraging. However, several devices are only available for open surgery and cannot be inserted endoscopically, while others are small enough but expensive and not easily available. Therefore, we developed a novel simple suture guide device, which can be applied endoscopically while being relatively low-cost.

## Methods

### Interventions

This study was approved by the Ethics Committee of The Affiliated Hospital of Medical School of Ningbo University (KY20201112).

### Annulus closure devices (ACDs)

**FIGURE 1:**
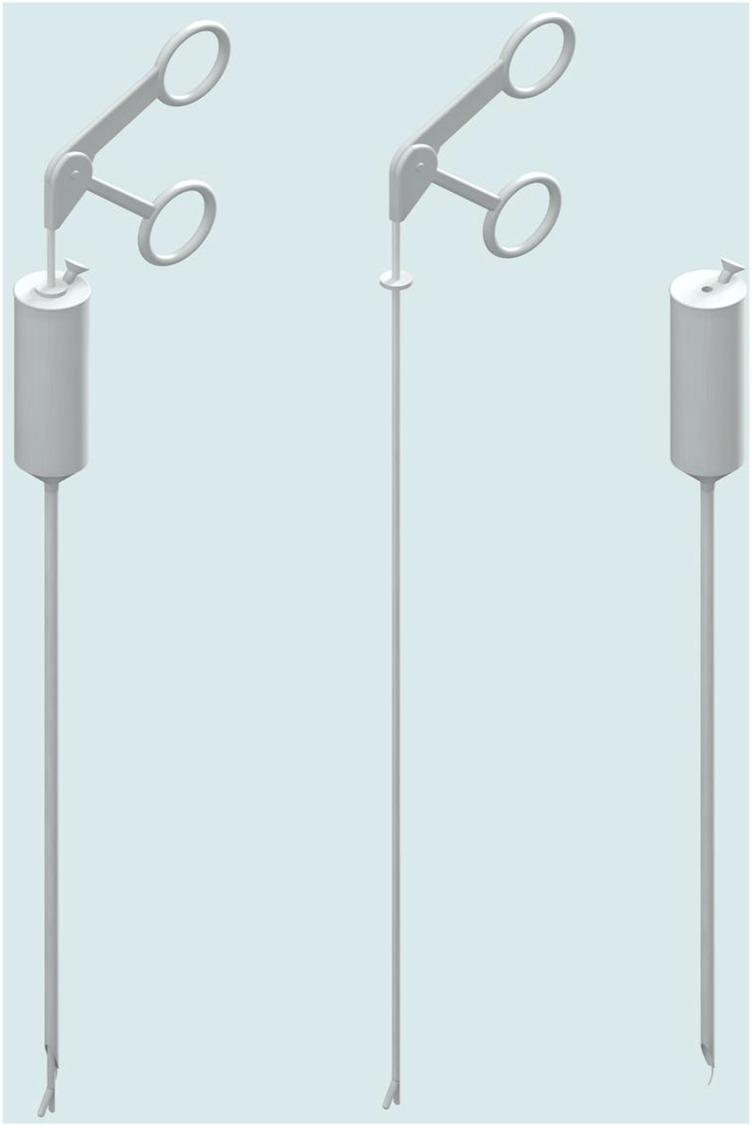
The 3D design drawing of ACD.

In this study, we utilised an ACD fabricated by our research group (Figure 1). The ACD is divided into the grip, the needle body and the puncture head. The handle and needle body are made of 304 stainless steel with a length of 200 mm. The diameter of the internal sewing channel is 0.75 mm, and the diameter of the wire pick-up pliers channel is 2.8 mm. The length of the matching wire pick-up pliers is 280 mm.

### Suture procedures

**FIGURE 2:**
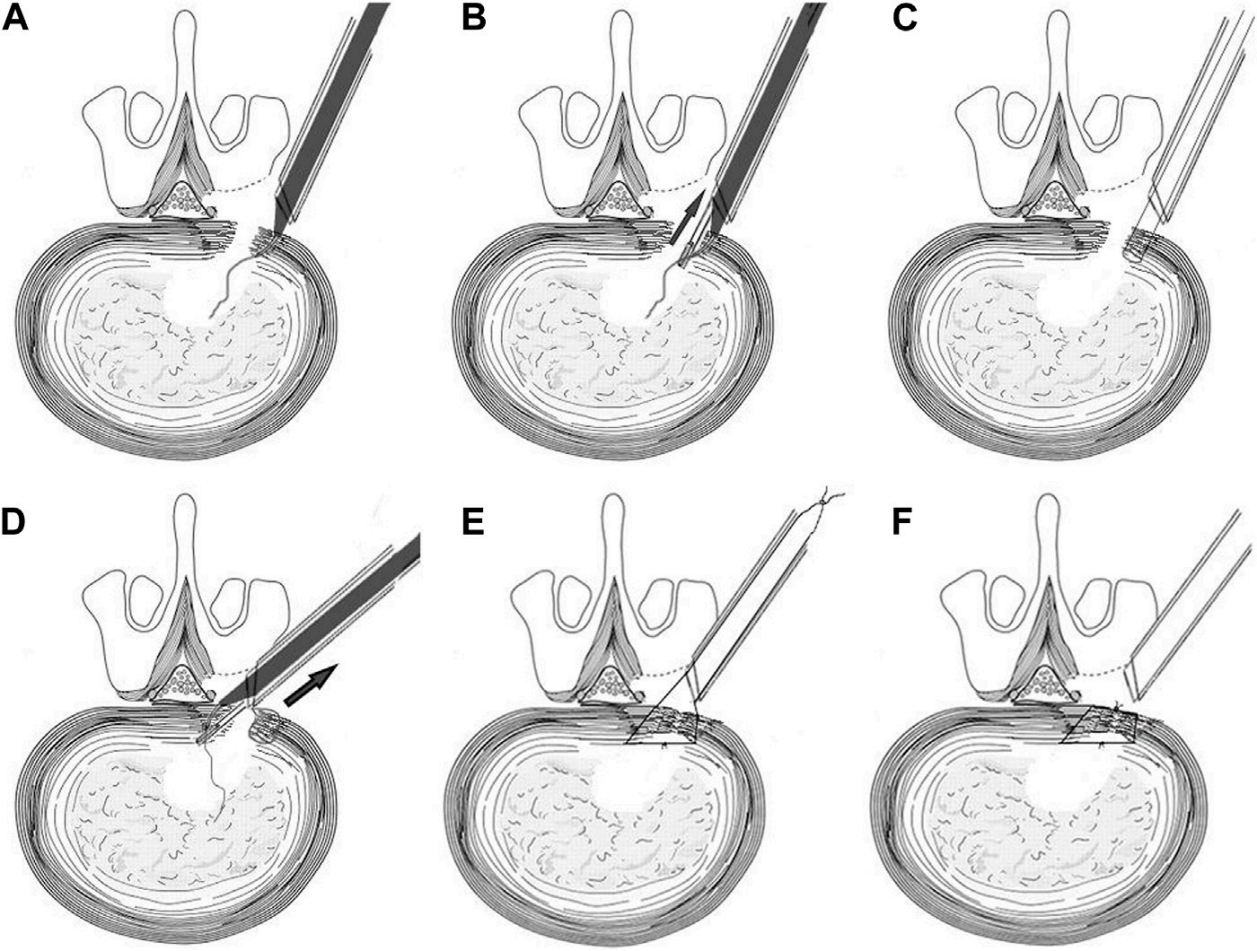
Schematic diagram of the suturing procedure. **(A)** puncture; **(B,C)** clip and pull the suture line; **(D)** repeat on the contralateral side; **(E,F)** knot.

**FIGURE 3:**
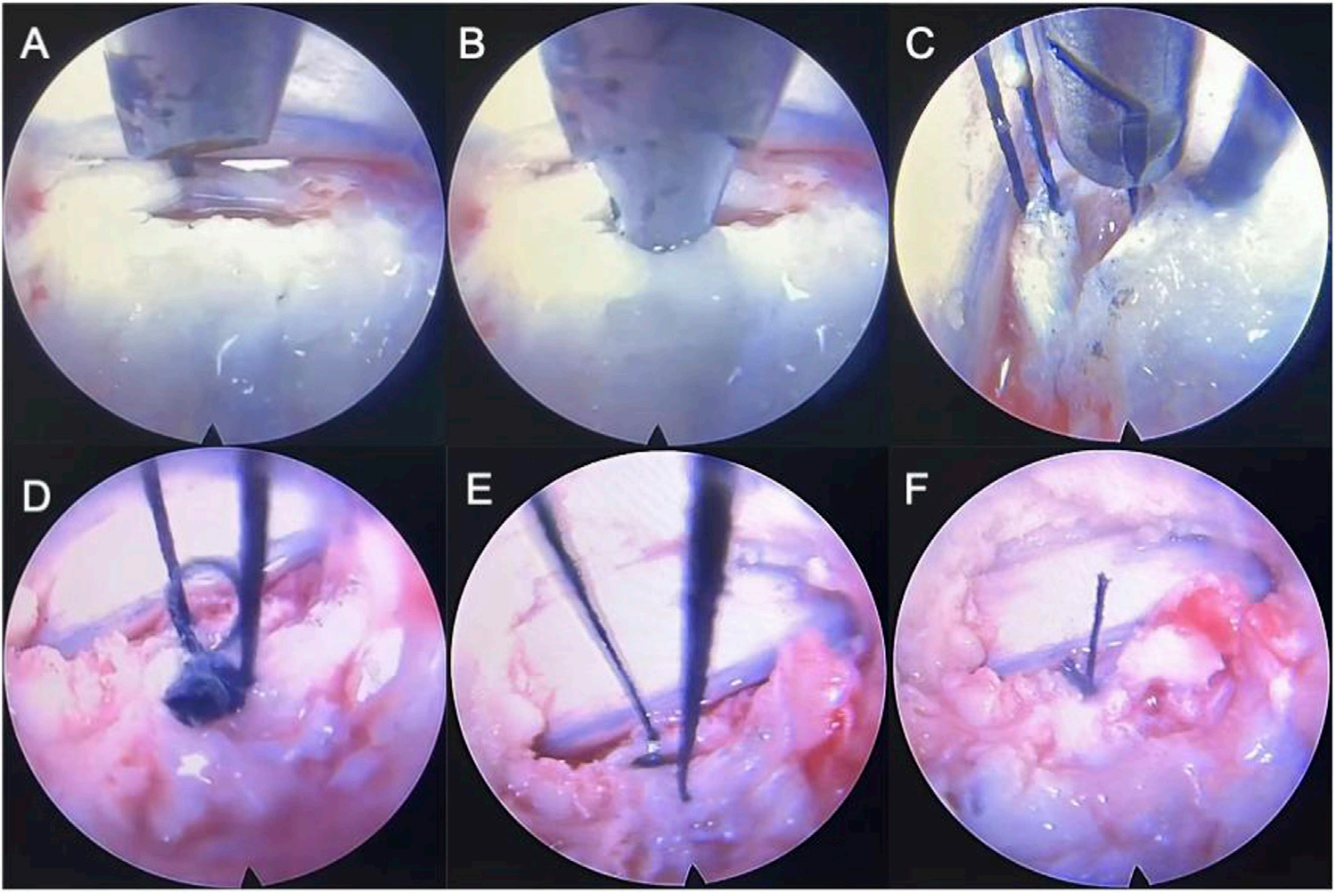
Simulation of a fully endoscopic suture of sheep IVD model. **(A)** puncture; **(B)** clip and pull the suture line; **(C)** repeat on the contralateral side; **(D,E)**, **(F)** knot the middle two lines, pull the outer two lines to bury the knot into the disc, then knot the outer two lines, and use a long push knotter to push the knot in and tighten it.

1) Placement of suture lines: 3–0 suture silk thread (length >400 mm) is moistened and then sucked into the suture channel of the ACD with a negative pressure suction device. Reserve the thread length of 10–15 mm at the tip of the puncture needle.
2) Placement of ACD: Insert the ACD through the working channel of the microendoscopic so that the puncture needle is located in the scope view.
3) Puncture: Puncture the tip into the edge of one side of the broken AF under direct vision and push the ACD to enable the puncture needle and suture to enter the disc.
4) Clip and pull the suture line: Grasp the suture line with the wire pick-up pliers and pull it out. Exit the ACD, allowing both ends of the suture line to lie outside.
5) Repeat on the contralateral side: The ACD reload the lines and repeats the above suturing operation on the contralateral edge of AF.
6) Knot: Knot the middle two lines, pull the outer two lines to bury the knot into the disc, then knot the outer two lines, and use a long push knotter to push the knot in and tighten it.
7) Adjustment: If the AF rupture is too large or the effect of a single suture is unsatisfactory, the above suture steps might repeat to achieve multiple sutures (Figure 2; Figure 3).

### Sheep-isolated intervertebral disc models

Obtain healthy, similarly sized sheep lumbar vertebrae, amputate the posterior column structures, and excise excess paravertebral soft tissues. The vertebral body is cut transversely in the middle of the vertebral body with a chainsaw and divided into separate functional discal units (FDU). The FDU consisted of the lower vertebral body of the previous vertebral body, the IVD, and the upper vertebral body of the next vertebral body. The AF was incised in the anterolateral aspect of the FDU and divided into the Control group (Group C) (incised discs with no suturing), Hand stitching group (Group H) and Suture device stitching group (Group S) with 10 in each group for a total of 30.

**FIGURE 4:**
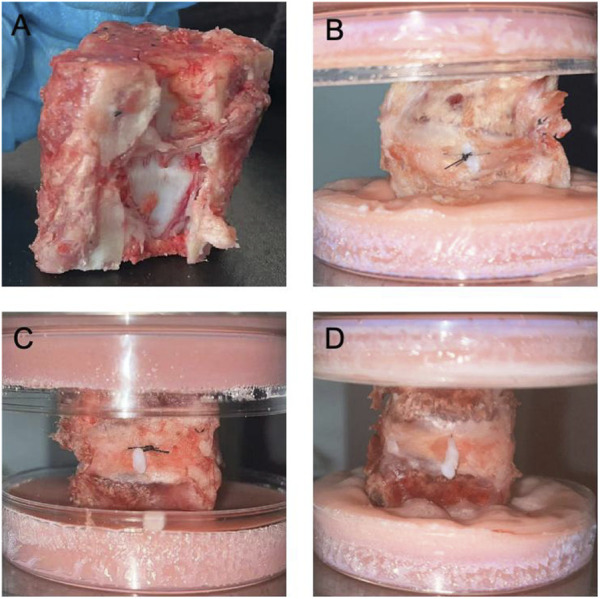
Sheep-isolated IVD models and NPs leakage after compression in respective groups. **(A)**. Functional Discal Unit (FDU); **(B)**. Suture device stitching group (Group S); **(C)**. Hand stitching group (Group H); **(D)**. Control group (Group C).

Following completion of the suture operation, the FDU was placed in a mould to fix the upper and lower vertebrae with dental powder to ensure uniform force. Record the load-displacement curves, failure load readings (peak readings) and NP leakage (Figure 4).

### Statistical analysis

Statistical analyses were performed with SPSS Version 26 (IBM Corporation, Armonk, NY). Kruskal Wallis test was performed to compare the failure load of each group, and *p* < 0.05 was considered statistically significant.

## Results

This study included 30 FDU models. The difference in failure load between Group H and Group C was statistically significant (*p* = 0.033); Group S and Group C had a statistically significant difference in failure load (*p* < 0.001) (Table 1; Table 2).

**TABLE 1:** Failure load pressure of Group C and Group H in sheep-isolated intervertebral disc model.

Index	Group C	Group H	Normality	Homogeneity of var	P.method	*p*.value (Adj)
Failure load N) Mean (±SD)	188.81 ± 32.56	966.18 ± 148.26	Yes	No	Kruskal–Wallis	0.033

**TABLE 2:** Failure load pressure of Group C and Group S in sheep-isolated intervertebral disc model.

Index	Group C	Group S	Normality	Homogeneity of var	P.method	*p*.value (Adj)
Failure load N) Mean (±SD)	188.81 ± 32.56	1889.80 ± 220.72	Yes	No	Kruskal-Wallis	<0.001

## Discussion

With further research, closure of the AF notch is increasingly recognised as a valuable method of preventing IVD herniation after discectomy (Klassen et al., 2017; Thomé et al., 2018; Ardeshiri et al., 2019). AF repair aims to preserve the remaining NP tissue, minimise the recurrence of IVD herniation, and maintain the water content and pressurisation of the NP tissue. An annular suture reduces early postoperative recurrence, maintains the intervertebral space’s height, reduces the nerve root’s mechanical and inflammatory irritation, and promotes scar healing in AF (Parker et al., 2016; Li et al., 2020).

Previous AF closure strategies include mesh-like devices, sutures (Heuer et al., 2008), and patch and plug-like implants (Bron et al., 2010; Chik et al., 2013). However, these are not effective in closing AF defects. The ideal AF repair solution is tissue-engineered materials to promote AF regeneration (Du et al., 2020; Shamsah et al., 2020; Wang et al., 2020), but there are several drawbacks: 1) it is currently stuck in the experimental stage; 2) the clinical application faces various safety and ethical issues; 3) the practical application is still pending. The ACDs that have been applied in clinical practice are The Xclose Tissue Repair System, The AnchorKnot^®^ suture-passing device, The Barricaid^®^ Annular Closure Device, Beijing 2020 Medical Science and Technology’s Disposable Fibre Loop Suture Device (EFIT-I-II-III-IV-V, ELAS-A, SMILE, STAR). The Xclose Tissue Repair System decreased the risk of re-herniation and re-operation, favouring the short-term outcome of the patient (2 years) with no additional increase in surgical risk. However, the symptoms of back and leg pain caused by the surgery were significant (Bailey et al., 2013; Choy et al., 2018). Suturing the AF with The AnchorKnot^®^ suture-passing device significantly reduced the rate of re-herniation; however, it was not effective in maintaining the volume of the IVDs (Bateman et al., 2016). The Barricaid^®^ Annular Closure Device restores IVD height, reduces pain and decreases re-herniation rates without increasing the risk of epidural haematoma (Bailey et al., 2013; Klassen et al., 2017). Nevertheless, it carries risks of device prolapse, inflammation and osteophyte formation (Parker et al., 2016; Strenge et al., 2019; Miller et al., 2020). Our ACD features the following advantages: the device is designed to be utilised in minimally invasive total endoscopic spine surgery with no foreign body residue in the body except for the suture lines; Convenient and affordable, suitable for areas where healthcare costs are controlled, or other ACDs are not available.

Our study demonstrated the technical feasibility of a novel ACD for repairing AF. In the sheep IVD *ex vivo* experiments, the mean failure load of Group H and Group S were significantly greater than those of Group C (*p* = 0.033, *p* < 0.001). However, the mean failure load of Group H was less than that of Group S. The reason is that the Group H suture uses a rounded needle, which requires a shallow needle insertion as it will not be able to return the needle if inserted too deeply. With the long tip of the ACD, the Group S suture will entail deeper penetration of the needle, which will suture more AF tissue and increase AF strength.

There are some limitations in our study. 1) we only performed simple biomechanical assessments. 2) the reason for utilising sheep IVDs in this study is that they are biomechanically similar to human IVDs and have been utilised as an *in vivo* or *in vitro* model of IVD degeneration (Hoogendoorn et al., 2008; Paul et al., 2013). Nevertheless, there are still differences, such as the sheep IVD containing significantly less NP than the human IVD. In the pressure test, many models failed to cause NP leakage when the pressure reached the maximum range of the instrument owing to the low NP content. 3) we applied the single-axis load, failing to fully correspond to the normal situation. The reason is that torsion, shear and buckling also occur during the usual walk (Smit et al., 1997; Smit, 2002). And uniaxial compression is unlikely to cause herniation under physiological or even supraphysiological loading conditions (Berger-Roscher et al., 2017). Our next research needs to refine the multiaxial mechanical testing. On the other hand, we utilised continuous rather than cyclic loading at a specific amplitude and frequency, which is at variance with the IVD pressures of daily human behaviour. 4) the simple suture apparatus features a complicated suturing procedure requiring a high-level learning curve.

## Conclusion

This study provides reasonable reasons to believe that the simple suture guide device described here is technically feasible for AF defect closure. It thus constitutes an encouraging proof of concept for the proposed device; however, it does not constitute a complete demonstration of the device’s feasibility in the clinical setting considering that the annulus closure operation is performed *ex vivo* on functional spinal units, as opposed to within an environment that mimics the clinical setting. To this end, confirmatory experiments will be conducted such as more multiaxial or dynamic mechanical testing, and notably performing the surgery on sheep models instead of on *ex vivo* functional spinal units.

## Data Availability

The raw data supporting the conclusion of this article will be made available by the authors, without undue reservation.
